# Therapeutic Approaches of Botulinum Toxin in Gynecology

**DOI:** 10.3390/toxins10040169

**Published:** 2018-04-21

**Authors:** Marius Alexandru Moga, Oana Gabriela Dimienescu, Andreea Bălan, Ioan Scârneciu, Barna Barabaș, Liana Pleș

**Affiliations:** 1 Department of Medical and Surgical Specialties, Faculty of Medicine, Transilvania University of Brasov, Brasov 500019, Romania; moga.og@gmail.com (M.A.M.); dr.andreeabalan@gmail.com (A.B.); urologie_scarneciu@yahoo.com (I.S.); 2 Department of Fundamental Disciplines and Clinical Prevention, Faculty of Medicine, Transilvania University of Brasov, Brasov 500019, Romania; barabas.mihail@gmail.com; 3 Clinical Department of Obstetrics and Gynecology, The Carol Davila University of Medicine and Pharmacy, Bucharest 020021, Romania; plesliana@gmail.com

**Keywords:** botulinum toxin, chronic pelvic pain, overactive detrusor, vaginism

## Abstract

Botulinum toxins (BoNTs) are produced by several anaerobic species of the genus *Clostridium* and, although they were originally considered lethal toxins, today they find their usefulness in the treatment of a wide range of pathologies in various medical specialties. Botulinum neurotoxin has been identified in seven different isoforms (BoNT-A, BoNT-B, BoNT-C, BoNT-D, BoNT-E, BoNT-F, and BoNT-G). Neurotoxigenic Clostridia can produce more than 40 different BoNT subtypes and, recently, a new BoNT serotype (BoNT-X) has been reported in some studies. BoNT-X has not been shown to actually be an active neurotoxin despite its catalytically active LC, so it should be described as a putative eighth serotype. The mechanism of action of the serotypes is similar: they inhibit the release of acetylcholine from the nerve endings but their therapeutically potency varies. Botulinum toxin type A (BoNT-A) is the most studied serotype for therapeutic purposes. Regarding the gynecological pathology, a series of studies based on the efficiency of its use in the treatment of refractory myofascial pelvic pain, vaginism, dyspareunia, vulvodynia and overactive bladder or urinary incontinence have been reported. The current study is a review of the literature regarding the efficiency of BoNT-A in the gynecological pathology and on the long and short-term effects of its administration.

## 1. Introduction

The incidence of chronic pelvic pain in women is constantly increasing, and is approximately 15% worldwide [1]. Jarell et al. [2] defined chronic pelvic pain as pain not related to gastrointestinal problems, menstruation or sexual activity, with a complex etiology. Chronic pelvic pain affects both women and men through common mechanisms, involving the central nervous system. The result is a regional pain syndrome that affects the entire pelvis. The triggers may be relatively benign, but individuals predisposed to chronic pelvic pain syndrome develop a series of sensory abnormalities and may perceive normal sensations as increased, until the point of unbearable pain and dysphoria. It is also associated with psychological, sexual, social and behavioral problems [2,3]. Other sequelae of this pathology include: decreased physical activity, impairment of social and family relationships, depression and accompanying vegetative signs such as sleep and appetite dysfunctions [4]. Potentially beneficial drugs include medroxyprogesterone depot or hormone therapy, but only in association with behavioral therapy [5,6]. Several treatments including conventional or homeopath drugs have been proposed for the management of this pathology. Medicinal plant supplements are therapeutic alternatives, when traditional interventions (surgery, anti-inflammatory or antalgic medication) fail to manage the disease [7]. Medicinal herbs have various effects on the women reproductive system, being used worldwide, in several pathologies including vulvodynia, vaginism, chronic pelvic pain of unknown etiology or urinary tract pathologies [3]. A study conducted in six European countries in 2016 pointed that several plants, such as *Plantago psyllium*, *Prunus Africana* or *Equisetum arvense*, could be used in the treatment of chronic pelvic pain of gynecological or urinary origin. Even if medicinal herbs are frequently used worldwide, new studies are mandatory to propose new drugs [7].

Another alternative to the treatment of several gynecological diseases, more studied nowadays, is Botulinum toxin (BoNT). Botulinum Toxin A (BoNT-A) has been used to treat various gynecological pathologies such as: chronic pelvic pain, vaginism, dyspareunia and urinary incontinence with overactive bladder or sphincter dyssynergia. This article is a review of the current published data regarding the administration of BoNT in gynecological pathology, but, to recommend the wider use of this treatment, it is essential to carry out more research. BoNT/A1 and BoNT/B1 are the only BoNT types used for clinical purposes and BoNT-A is the most studied isoform for therapeutic purposes. Clinical trials on this topic have defined the safety and tolerability profile of BoNT-A [8]. All patients from the clinical studies injected with BoNT-A understood the possible undesirable effects of the treatment, giving their approval through signing the informed consent [9]. The incidence of adverse effects was observed to be approximately 25% in the BoNT-A treated groups compared to 15% in the control group. Among the side effects of the treatment with BoNT-A, the most frequently mentioned was the focal weakness, erythema, edema or hyperesthesia [10]. BoNT-A is used in various fields of medicine: dermatology, motion disorders, ophthalmic disorders, and gastrointestinal disorders, as well as in urogynecology pathologies, having high efficiency with minimal adverse effects. Table 1 summarizes the clinical applications of BoNT in medicine [11].

Regarding the use of other serotypes, such as BoNT-B, it has been used to induce human muscle paralysis but, according to Sloop and coworkers’ research, the paralysis resulting from BoNT-B is not as efficient as the one resulting from BoNT-A [12].

## 2. Botulinum Toxin

BoNT are proteic neurotoxins produced by anaerobic sporulated bacteria of the *Clostridium* genus. Pirazzini et al. [13,14] described in a comprehensive review four different clostridial groups (Clostridium *Botulinum* groups I–IV, *Clostridium baratii* and *Clostridium butyricum*) that are known to produce the seven serotypes of BoNTs (BoNT-A to BoNT-G) [15]. Based on the amino-acid sequences, the serotypes are divided into subtypes, being more than 40 BoNT subtypes identified [16].

The first neurotoxin serotypes were identified in 1919 (BoNT-A and BoNT-B) and the last one in the year 1969 (BoNT-G) [16,17,18]. In 2017, Zhang et al. described a new BoNT-serotype (BoNT-X) [19]. The particularity of this serotype is that can cleave VAMP4 (which mediates vesicle fusion between endosome and TGN) [20,21] and Ykt6—an atypical SNARE without transmembrane domain (an essential protein in yeast, involved in membrane fusion events such as ER-Golgi, intra-Golgi, autophagosome formation) [22]. Zornetta et al. [23] also described in 2016 the first non-Clostridial botulinum like toxin (BoNT-Wo) identified from *Weissella oryzae* (an anaerobe isolated from fermenting rice) [24]. The particularity of this toxin is that it cleaves the VAMP at a single site (a unique Trp-Trp peptide bond, localized within the juxtamembrane segment of VAMP). Recently, Zhang et al. reported another Botulinum Neurotoxin-like toxin in the *Enterococcus faecium* strain isolated from cow feces (BoNT-En) [25]. BoNT-En cleaves two proteins that mediate synaptic vesicle exocytosis in neurons: SNAP-25 and VAMP-2.

BoNT consists of two chains: a heavy chain of 100 kDa and a light chain of 50 kDa linked by a disulfide bond which is extending from the heavy chain and surrounds the light chain like “a belt” [26]. The heavy chain contains a N-terminal HC that mediates the translocation of the LC (which acts as a protease, cleaving various proteins of BoNT-A, -C and -E cleave SNAP-25; BoNT-B, -D, -F and -G cleave VAMP1, -2 and -3; and BoNT C cleaves syntaxin 1) into the endosomal membranes. The cleavage of one of the three SNARE proteins prevents neurotransmitter release from neurons by blocking the fusion of synaptic vesicles to plasma membranes [13,27,28]. Table 2 summarizes the BoNT serotypes, subtypes, the target SNARE proteins, and their intracellular compartments.

**Figure 1:**
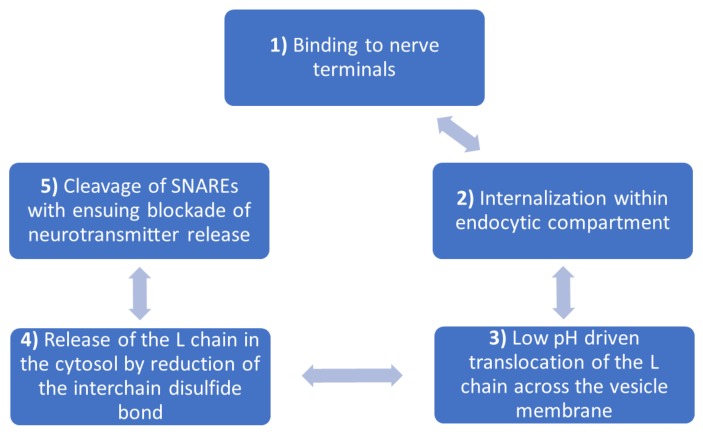
The five steps of BoNTs’ mechanism of action inside nerve terminal.

After exceeding the intestinal barrier, BONTs spread into the extracellular fluids, entering the lymphatic system, followed by spreading into the blood circulation [29], without crossing the blood barrier. BoNTs can bind to any neurons, but they are distributed primarily to the peripheral nerve terminals [14]. The molecular mechanism of BoNTs inside nerve terminals is described in Figure 1.

The first step is binding to the presynaptic vesicle membrane of the nerve terminals, through HC domain, to two independent receptors: a PSG receptor and a protein receptor of the synaptic vesicle (glycosylated SV2 in case of BoNT-A1 and BoNT E1, synaptotagmin I/II for BoNT-B1, BoNT-DC and BoNT-G) [30,31,32,33,34]. The next step involves internalization of BoNT, through dual binding with synaptic vesicle receptors and PSG. Following this process, the strength of BoNT interactions with the membrane increases [13,14,33].

The third step of the process, namely translocation has been extensively studied. The vesicular ATPase proton pump generates a transmembrane pH gradient, to translocate the L-chain from the synaptic vesicle into the cytosol. ATPase inhibitors are an important component, because they block completely the nerve termination intoxications by BONTs [35,36,37,38,39,40]. After translocation, the L chain is released on the cytosolic side of the membrane. However, this process requires the inter-change disulfide bond to be reduced, because the BONTs that possess reduced inter-chain disulfide bonds do not form channels. Fisher et al. described in their paper the importance of reduction in the inter-chain disulfide bon that needs to take place at any stage before the exposure to the cytosol. It is necessary because it prevents the L-chain translocation. Several enzymatic systems (thioredoxins and glutaredoxins) are involved in the reduction of protein disulfide bond, having a major role in the release of L chain into the neuronal cytosol [41]. After the enzymatic system reduction of the disulfide bond, the toxin can interact with the target proteins [40,42,43,44,45].

The final step of the BoNTs mechanism is the cleavage of SNARE proteins with ensuing blockade of neurotransmitter release. The L chains of all BoNTs are specific metalloproteases for one of the SNARE proteins: VAMP, SNAP25 or syntaxin. BONT-A and -E cleave SNAP25; BONT-B, -D, -F, and -G cleave VAMP; and BONT-C targets syntaxin and SNAP25. The result of the proteolytic actions is the prolonged inhibition of the neurotransmitter release, followed by neuroparalysis [13,46,47].

**Figure 2:**
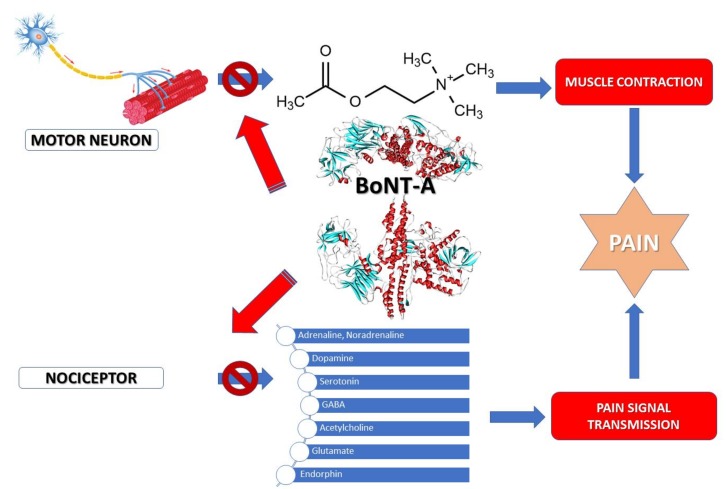
Mechanism of action of BoNT-A in pain. Inhibition of acetylcholine and neurotransmitter released from motor neuron and nociceptor by BoNT-A reduces pain by inhibiting the pain signal transmission.

BoNT-A is used in medicine in a wide range of muscular dysfunctions because it acts on nerve endings and inhibits the release of acetylcholine in synaptic spaces, preventing muscle spasm [48]. BoNTs acts on chronic pain, spasm and dystonia and could be successfully used to relieve these symptoms [49,50]. Of all BoNT serotypes, BoNT-A specifically cleaves SNAP-25 and thus prevents the release of acetylcholine in the synaptic space. As the synapses are blocked by the action of the toxin, the neuron will form new ones, a process known as sprouting [26,51]. A systematized description of the mechanism of BoNT-A in pain inhibition can be observed in Figure 2.

## 3. Review of the Literature Regarding the Gynecologic Indications for the Use of BoNT-A

### 3.1. Use of BoNT-A in the Treatment of Vaginism

The term “vaginism” describes the involuntary, recurrent or persistent contraction of the perineal muscles that surround the outer third of the vagina. It occurs during sexual intercourse and/or penetration with a swab or vaginal speculum during a gynecological examination. This involuntary contraction of the perineal muscles can aggravate or even make sexual life impossible [52].

The severity of vaginism could be classified according to the Lamont Scale, depending on the presenting symptoms and pain during the gynecology exams. Vaginismus was first described in 1978 by Lamont, who divided the pathology into four degrees [53]:

- 1st degree:
  o Levator and perineal spasm relieved with reassurance
  o Able to tolerate vaginal exam
- 2nd degree:
  o the perineal spasm is maintained through the gynecology exam
  o Unable to relax for the pelvic exam
- 3rd degree:
  o Spasm of the levator muscle
  o Elevation of buttocks to avoid the gynecology exam
- 4th degree:
  o Perineal and levator spasm
  o Adduction of thighs, elevation of buttocks, unable to tolerate the pelvic exam.

Depending on the severity degree, there are cases when penetration with vaginal swabs or vaginal speculum is allowed, but, in severe cases, penetration with any gynecological instrument or sexual intercourse is impossible [54]. Kegel exercises, relaxation and physical examination are considered the first line treatment in this pathology. In addition, voluntary control of perineal muscle contraction is a key factor in the successful treatment of vaginism [53]. Anxiolytic therapeutic agents and local treatments (lubricants and anesthetic creams) have been used as pharmacological treatments of this pathology, but approximately 10% of patients do not find amelioration in the symptoms [54].

Because of the numerous unresponsive cases to the conventional treatment, several authors investigated the effect of BoNT in this pathology [54,55,56,57]. The first case of vaginismus treated with BoNT-A was described in 1977 [58] and since then it had been carried out multiple studies.

A retrospective study from 2004 included 24 women aged 19–34 years with 3^rd^- or 4th-degree vaginism, without previous treatment [54]. Prior to injection, 500 U BoNT-A was diluted with 1.5 mL of saline solution and a total dose of 150–400 U was injected equally into levator ani muscles, in three points on each side, under sedation with Midazolam and with Oxygen administration. For the first cases, 150–200 U BoNT-A was used, with the dose gradually increased for the following patients, up to 400 U. Patients were monitored on average for 12 months and the conclusions showed that: 95.8% of the patients did not show any resistance or showed reduced resistance to post-injection vaginal examinations, 75% achieved satisfactory sexual intercourse after the first injection and 16.7% had mild pain at penetration after the first injection. In addition, recurrent vaginism in patients treated with BoNT-A was not detected.

In a case-control study, the efficiency of BoNT use in the treatment of vaginism was investigated [55]. The cohort consisted of thirteen cases of women diagnosed with vaginism, with an average age of 26.6 years. The cohort was divided into two groups: eight patients suffering from vaginism and five patients diagnosed with vaginism prior to the treatment, considered the control group. The first group was injected with 25 U of BoNT diluted in 1 mL of saline in each bulbospongiosus muscle. The controllers were injected with saline solution. After the injection, the patients were followed for an average of 3.3 months. The results obtained were encouraging: improvements were observed, and, in all cases, sexual life became possible or satisfactory. However, recurrences of vaginism have also been reported. There were no improvements in the control group.

To point out the utility of BoNT-A in the treatment of vaginismus secondary to vulvar vestibulitis syndrome, Bertolasi et al. recruited 39 women whose electromyography (EMG) recordings in the levator ani muscle had showed reduced resting and reduced inhibition during exercise [56]. The patients were injected with BoNT-A into repeated cycles under the guidance of EMG and were followed for an average of 105 (±50) weeks. Four weeks after each cycle, the women underwent EMG evaluations, vaginal examinations, evaluation of bowel and bladder symptoms completed VAS and the female sexual function index scale (FSFI). The results of the questionnaires were satisfactory at the first follow up (at four weeks after the first injection of BoNT-A) and the results maintained, with the increase in the number of subsequent injections. At the end of the follow-up period, 63.2% of the patients were completely cured, 15.4% requested re-injections and 15.4% dropped out the study before finishing it.

Another retrospective study that pointed out the use of BoNT-A in the treatment of vaginism was conducted on a cohort of 20 patients that have been treated with BoNT-A injections during 2005–2009 [57]. The patients were divided according to the severity of vaginism: 12 women with primary vaginism, 5 women with secondary vaginism and 3 women with severe dyspareunia. Initially, low doses of BoNT-A were used, and then the doses increased from 100 U to 150 U, diluted in 2 mL saline solution, injected under sedation (15–20 mL of bupivacaine 0.25% with epinephrine 1: 200.000) in several points along each side of the vagina (into the bulbocavernosus, pubococcygeus and puborectalis muscles). At the time of the study, 16 patients managed to have sexual intercourse in two weeks to three months after the injection and a patient was considered a failure because not even the smallest penetration dilator could ever be used. Patients continued to have a low degree of discomfort and vaginal burns during early sexual intercourse attempts, but this problem disappeared within a few weeks of completing the treatment with BoNT-A.

Table 3 summarizes the clinical studies regarding the use of BoNT-A in the treatment of vaginism.

### 3.2. Use of BoNT in the Treatment of Vulvodynia

Vulvodynia is a sexual dysfunction manifested by vulvar pain and orgasmic difficulties that cause a difficult sexual life. Women affected by this pathology receive only symptomatic treatment, anti-inflammatory and analgesic drugs, while psychotherapy can treat the fear of pain [60]. In modern medicine, BoNT could be used to treat this pathology when other treatments fail. It acts through a permanent neuromuscular blockage and muscle function recovery is achieved by forming new neuromuscular junctions [61]. The etiology of vulvodynia is not fully known, although it has been extensively researched. The factors involved in this pathology could be: inflammatory, genetic, infectious, hormonal or mechanical [62,63]. These factors induce modifications through three different pathways: sexual function, nervous system pain and pelvic floor muscles [64].

Yoon et al. performed a study regarding the use of BoNT-A in the treatment of vulvodynia [60]. The cohort consisted of seven women with genital pain that were injected with 20–40 U BoNT-A. All patients reported that the pain decreased after injections and the subjective pain score improved from 8.3 to 1.4, with no recurrences (the follow-up period was 4–24 months) Patients have also reported that, after treatment, no significant pain or discomfort occurred during or after sexual intercourse. In 2009, Petersen et al. evaluated in a randomized, double blinded, placebo-controlled study, the efficacy of BoNT-A injection in 32 women with vulvodynia and compared the results with a control group of 32 women [63]. Twenty units of BoNT were diluted in 0.5 mL saline solution and injected into bulbospongious muscles, while, for control cases, 0.5 mL saline solution was used. Both groups achieved a significant reduction in pain and the conclusion was that BoNT-A did not reduce the pain, does not improve sexual function and does not influence the quality of life compared to the placebo group.

Vulvodynia is a syndrome defined by a sharp pain in the vulva that does not have a well-defined cause which makes it very difficult to treat. The most common clinical form of vulvodynia is the provoked vestibulodynia, also named vulvar vestibulitis [65]. BoNT-A administered in high doses appears to have found utility in the treatment of this pathology that does not have a known organic substrate. Pelletier et al. administered BoNT-A to a group of 20 women aged 18–60 with provoked vulvodynia [66]. Each patient was injected with 50 U of BoNT-A into the bulbospongious muscles under EMG guidance. After three months, 80% of patients confirmed a decrease in pain intensity, and quality of life and sexual life improved significantly in the first six months. A retrospective study compared the effects of different doses of BoNT-A and Gabapentin in patients with vulvodynia also concluded that the symptoms of the patients injected with BoNT-A, measured through VAS scale were significantly improved in the group treated with Gabapentin (an anti-epileptic drug, that is also used to treat neuropathic pain) [67,68]. In Table 4 are summarized the clinical studies regarding the use of BoNT-A in the treatment of vulvodynia.

One of the causes of the occurrence of vulvodynia is the aberrant increase in the number of nociceptors. Intraepithelial neural hyperplasia associated with hypersensitivity of peripheral nociceptors generates a strong pain in the vestibule. BoNT was successfully used in this pathology. The pain is released through blocking the release of acetylcholine from parasympathetic neurons and from sympathetic post-ganglionar neurons. BoNT has been also used, with a beneficial effect on dyspareunia. These effects could be explained by two theories: the first refers to the decrease of the pelvic muscular hypertonicity and implicitly of the pain, by paralyzing the musculature. The second mechanism is the blockade of neurotransmission at nociceptive receptors in the submucosal layers of the vestibule [69].

### 3.3. Use of BoNT-A in the Treatment of Chronic Pelvic Pain

A persistent pelvic pain, lasting more than six months, which can be conceptualized as a syndrome of somatic functional pain or as a regional pain syndrome defines chronic pelvic pain [5].

One of the causes of chronic pelvic pain syndrome is the spasm of the pelvic muscles, especially the spasm of the levator ani. Myofascial pain and spasm are defined as regional muscular pain characterized by the presence of trigger points. These are hypersensitive points distributed on the levator ani surface, which once touched, cause pain. The pain resulting from reaching these trigger points appears to result from the excessive release of acetylcholine and other neurogenic inflammatory substances from the neuromuscular junction. The management of pelvic floor muscle spasm requires a multidisciplinary approach and treatment strategies including the use of steroids, non-steroidal anti-inflammatory drugs, muscle relaxants, antidepressants, neuromodulators, selective norepinephrine reuptake inhibitors and injection of various substances into the triggering points such as local anesthetics, steroids and BoNT [70,71,72].

Adelowo et al. designed in 2013 a retrospective study on a cohort of 31 patients to evaluate the role of injections with BoNT-A in the levator ani muscle in women with refractory pelvic myofascial pain [73]. The pain was assessed during palpation of the pelvic floor muscles using a scale of 0 to 10, 10 being the most severe pain possible. Patient reassessment occurred before six weeks after injection and again after ≥6 weeks post injection. Thirty-one patients met the eligibility criteria but two were lost during follow-up. Overall, 79.3% of the patients reported on the re-evaluation a pain relief, while 20.7% reported an improvement in symptoms. The conclusion of this study was that BoNT-A injection into the levator ani proved to be effective for women with refractory myofascial pelvic pain, with only a few limited side effects. In 2006 a double-blind randomized study was conducted to estimate the utility of BoNT versus placebo in women that have reported pelvic spasms and chronic pelvic pain lasting more than two years [74]. Thirty women were injected with 80 U of BoNT while 30 with saline solution in the pelvic floor muscles. Their subjective symptoms (dysmenorrhea, dyspareunia and pelvic pain of non-menstrual origin) were quantified with VAS scores (Visual analog scales). VAS is a measurement instrument used to document the symptoms severity in different patients and to assess the effectiveness of therapy in those patients [75]. The pain of the pelvic floor was measured by vaginal manometry. After six months, the patients were re-evaluated and the conclusion was that the reduction of pelvic spasm could reduce some types of pelvic pain. BoNT-A reduces pelvic floor hypertonia more than placebo, so it could be used in women with refractory pain.

Gajraj et al. presented a case of a 60-year-old woman presenting a four-year-long pelvic pain with leg irradiation and irradiation in the vagina and rectum, aggravated by clinostats [76]. The pain was assessed at a level of 4–8 out of 10 on the VAS pain scale. Physical examinations did not reveal any focal neurological signs. The vaginal examination showed sensitivity and tenderness in the right anterior and right posterior lateral regions. The patient was injected in the internal obturator muscle with 0.25% bupivacaine, resulting in a 90% reduction in pain for 12 h. Progressive and postprocedural mean scores on the VAS scale were 7 out of 10 and 1 out of 10, respectively. After a subsequent BoNT-A injection, the patient again reported a 90% decrease in pain for more than three months. In addition, after BoNT injection, there were no adverse effects such as motor weakness, and intestinal or bladder disorders. Therefore, BoNT-A has also shown its efficiency in this case.

Twelve women aged 18–55 years old, with objective hypertonia of the pelvic floor muscles for at least two years and chronic pelvic pain were recruited for testing the utility of BoNT [77]. Forty units of BoNT-A in three different dilutions were administered bilaterally in the puborectal and pubococcygeal muscles under conscious sedation. The results were favorable and uninfluenced by dilution. VAS pain scores improved for the cases with dyspareunia, but non-menstrual pelvic pain experienced insignificant reductions. At four weeks after treatment, it was observed a decrease with 37% in resting pressure measured through pelvic muscle manometer, reduction that decreased until 25% at 12 weeks. In addition, the quality of life scores improved.

A prospective study on women with refractory chronic pelvic pain and pelvic muscle spasm was conducted in 2015 to demonstrate the role of BoNT-A in the treatment of these dysfunctions [78]. BoNT-A injections in spastic pelvic muscles (up to 300 U) were performed through needle electromyographic guidance. Of the 28 women enrolled in the study, 21 qualified for analysis. The average age of the cases was 22–50 years and the comorbidities included interstitial cystitis/bladder pain syndrome in 42.9% of cases and vulvodynia in 66.7% of cases. Overall, 61.9% of subjects have reported improvement in the overall response assessment at four weeks and 80.9% at 8, 12 and 24 weeks post injection compared to baseline. Post-injection adverse reactions were also reported, including worsening of the following pre-existing conditions: constipation (28.6%), stress urinary incontinence (4.8%), fecal incontinence (4.8%) and urinary incontinence (4.8%). The conclusion of this study is consistent with the findings of other studies on this topic and suggests that BoNT could be useful in the treatment of pelvic floor muscle spasm and chronic pelvic pain, refractory to other therapies.

Levator ani syndrome is defined by chronic or recurrent episodes of rectal pain and affects approximately 6.6% of adults. There is no consensus on the pathophysiology of this painful syndrome, although the chronic hypertonia of pelvic floor muscles is the most common explanation [79]. Therefore, through the muscle spasm, the use of BoNT-A was attempted in the treatment of levator ani syndrome, however, without favorable results. Rao et al. conducted a randomized, placebo-controlled study on 12 cases with levator ani syndrome [80]. After BoNT-Administration into the anal sphincter, the duration and intensity of pain and the mean frequency remained unchanged compared to the baseline. The conclusion of the authors was that BoNT-A injections into the sphincter ani is secure but is not effective in relieving anorectal pain associated with this syndrome. Table 5 summarizes the clinical studies regarding the use of BoNT-A in the treatment of chronic pelvic pain and pelvic floor muscle spasm treatment.

Jhang J-F et al. [81] described in their research from 2015 a possible mechanism of BoNT-A on chronic pelvic pain. In several experimental studies involving both rats and humans, membrane receptors TRVP-1 and P2X3 have been observed to be up-regulated in the neuropathic pain. Xiao L. et al. concluded that a possible mechanism of BoNT-A is the reduction of TRPV-1 expression in spinal neurons of rats with hyperalgesia [82]. Muscular spasm seems to be usually associated with chronic pelvic pain [83,84] and the reduction of pain may also reduce the spasm. Myelinated and unmyelinated fibers (group III and IV, respectively) are nociceptors found in muscles, which can be sensitized by bradykinins, prostaglandin E, substance P, calcitonin gene-related peptide (CGRP) [85] and ATP [86]. BoNT-A is supposed to inhibit their release [85] and the activation of spinal cord neurons that are responsible for the pain transmission [87,88,89]. BoNT-A could also inhibit the spinal motor neurons alpha and gamma, mechanism through which pelvic muscle spasm could be stopped [85]. It is important to continue the research in this area and to investigate the mechanism of action and the effects of BoNT-A in the treatment of chronic pelvic pain.

### 3.4. Inferior Urinary System Dysfunctions

#### 3.4.1. Use of BoNT in the Treatment of Interstitial Cystitis (the Painful Bladder Syndrome)

BoNT-A injections into the urethral sphincter have been used in the treatment of lower urinary tract dysfunctions in the last 20 years to reduce bladder emptying, urethral and residual urine pressures [90,91]. The pathogenesis of interstitial cystitis is still uncertain, but there is a hypothesis that asserts that this pathology arises from neurogenic inflammation that activates bladder afferent nerves and causes bladder hypersensitivity [92]. Painful bladder syndrome is a clinical syndrome characterized by pain with supra-pubian localization caused by bladder filling associated with increased urinary frequency both day and night in the absence of proven urinary infection. There is currently no standard treatment [93], but there are studies in the literature that confirm the efficiency of BoNT-A in the management of interstitial cystitis, which has an antinociceptive effect on the visceral afferent nervous fibers [94].

Giannantoni A. et al. selected a group of seven women and instilled them with 200 U BoNT-A diluted in 100 mL of saline, without any anesthesia, to analyze the utility of this treatment in bladder painful syndrome [94]. The exclusion criterion for the cohort was detrusor overactivity. The solution was injected intravesical and maintained for 40 min and the results were evaluated after one week, one month and three months. However, the results were discouraging because short-term benefits were found for four of seven patients. The explanation consists in the molecular weight of BoNT-A that does not allow it to pass the epithelial barrier to the bladder to reach sub-urothelium and to act on nerve endings. Therefore, the intravesical instillation of diluted BoNTs is not an effective treatment in this pathology.

As the intravesical instillation of BoNT-A has not been found to be convenient, studies on this subject have further been conducted and it has been found that increased amounts of sensory fibers are located into the bladder trigonum. To evaluate the tolerability and efficiency of BoNT-A injections in patients with painful bladder syndrome that are refractory to standard treatment, Pinto et al. conducted a study on 17 patients (16 women and one man), which were investigated before treatment and one, three six, and nine months later [93]. All patients received 10 intra-trigonal injections with 10 U BoNT-A, diluted in 1 mL saline (a total of 100 U). The results were favorable: the pain level decreased, the urinary frequency decreased and O’Leary-Sant score increased. Therefore, the authors concluded that intra-trigonal injections with BoNT-A are useful in treating interstitial cystitis, also called painful bladder syndrome.

Satisfactory results were also obtained in the treatment of vesical dysfunctions, by injecting BoNT-A into the bladder wall [95]. Fifteen patients received 200 U BoNT-A diluted in 20 mL saline under general anesthesia and cystoscopic guidance. To evaluate the results, VAS pain scale and drainage charts were recorded, while urodynamic studies were realized before injections and at 1, 3, 5 and 12 months after the treatment. Overall, 86.6% of patients have reported an improvement after one and three months. In 26.6% of cases, at the five months follow up visit, it was observed that the effects persisted; nevertheless, the urinary frequency during day and night was increased. Twelve months after the treatment, the pain reappeared in all patients. Nine patients claimed dysuria one month after treatment.

Kuo et al. conducted a study on 10 patients to demonstrate the efficiency of suburothelial injection of BoNT-A in the management of chronic interstitial cystitis [96]. They injected 100 U of BoNT-A suburothelial in 20 places on five patients.Five other patients were injected with another 100 U of BoNT-A at the trigonal area of the bladder. However, the therapeutic results were disappointing because no favorable evolution was observed after three months of treatment. Carl et al. conducted a pilot study involving 29 patients with painful bladder syndrome and injected them with BoNT-A, to demonstrate that it is useful in the treatment of this pathology [97]. The toxin was injected submucosally, into the trigonal area, and the results contrary to those obtained by Kuo et al. [96], suggested that BoNT-A has an antinociceptive effect on the bladder afferent nerves in patients with chronic interstitial cystitis. The authors did not experience systemic side effects during and after treatment.

A single center, prospective, non-randomized study was conducted in 2007 by Ramsay et al. to evaluate the efficiency, tolerability and safety of BoNT-A when injected intravesical at patients with interstitial cystitis [98]. Eleven women with average age of 56 years were injected with BoNT-A. The conclusion of the study was that a significant symptoms reduction had been observed at about 10–14 weeks after injection. Another prospective study based on the safety and effects of BoNT-A repeatedly administered to patients with bladder painful syndrome, was conducted on a cohort of 16 patients [99]. They were exposed to four cycles of intratrigonal injections with BoNT-A. A second injection was administered at the three-month follow up visit. Complications such as urinary tract infections or bladder hypersensitivity were evaluated at different intervals. Improvement occurred after approximately 9.9 months (±2.4 months). No total remission of symptoms was seen in any of the patients and 5 of the 16 patients had uncomplicated urinary tract infections. The studies that are pointing out the usefulness of BoNT-A in interstitial cystitis treatment are summarized is Table 6.

Apostolidis et al. proposed a possible mechanism of action of BoNT in the treatment of detrusor activity, but further research is needed to determine the significant effects of BoNT-A in this pathology [100,101]. Karsenty et al. [102] conducted a study in 2008 that pointed out that BoNT-A could also action through inhibition of other neurotransmitters, receptors or neuropeptides. A review from 2016 [103] identified a possible mechanism for the effects of BoNT-A in the treatment of inferior urinary system dysfunctions. Several studies in vivo and vitro included in this research, highlighted that BoNT-A injections into detrusor could decrease the levels of capsaicin receptor TRPV1 and purinergic receptor P2X3 (whose expression is increased) in the suburothelial nerve fibers [104,105,106,107]. Patients with detrusor overactivity have shown increased densities of substance P and CGRP, according to Smet et al. [108]. Following those observations, several studies on animals identified a possible mechanism of action of BoNT-A, which consists of the reduced release of CGRP in rat model [109] and an inhibition of SP with reduced activation of P2X3 and TRPV1 receptors in suburothelium and detrusor muscle in guinea pig model [110], leading to peripheral denervation. However, future research must be conducted to clarify the proposed mechanism and the role of BoNT-A therapy in case of inferior urinary system dysfunctions.

#### 3.4.2. Use of BoNT-A in Urinary Incontinence through Neurogenic Overactive Bladder and Idiopathic Overactive Bladder

Urinary incontinence through both neurogenic (NOB) and idiopathic bladder hyperactivity (IOB) is a lower urinary tract dysfunction that affects many women with a prevalence of approximately 25% of the cases worldwide [111]. Bladder overactivity is defined by increased urinary frequency, feeling of urge and nycturia and could be associated with urinary incontinence. Anticholinergic therapy is essential in the treatment of overactive bladder but it also requires lifestyle and behavioral changes. If symptoms do not improve with anticholinergic therapy and lifestyle changes, urodynamic studies and cystourethroscopy are required [112]. Studies confirm the usefulness of intravesical injections with BoNT-A in women with overactive bladder (OB). After the injection of BoNT-A to the patients with neurogenic or idiopathic detrusor overactivity, there was observed a reduction in bladder detrusor pressure during both involuntary and voluntary contractions, which confirmed the BoNT-A influences the motor detrusor innervation. BoNT-A prevents the release of neurotransmitters such as Ach and in addition it acts on adenosine triphosphate (ATP), substance P and glutamate, decreasing the number of sensory receptors and nerves growth factor (NGF) in the bladder wall. These mechanisms of action may explain the utility of BoNT-A in the treatment of urinary incontinence through bladder overactivity [86].

During the period 2005–2009, 99 patients with OB were enrolled in a prospective, randomized, double-blind, placebo-controlled comparative trial [113]. Patients received a single injection with BoNT-A (50 U, 100 U or 150 U) into the vesical muscle. Three months after administration, a 50% improvement from baseline in urge and urinary incontinence was noticed. The 100 U and 150 U doses of BoNT were well tolerated and, in both cases, improvement was recorded. However, injections of 100 U showed reasonable efficiency with a lower post-voiding residual volume risk.

A comparative study between the response of patients with neurogenic detrusor overactivity and idiopathic detrusor overactivity to the first of administration of BoNT-A into the vesical detrusor was performed in 2005 by Popat et al. [114]. The study included 44 patients with neurogenic bladder overactivity and 31 with idiopathic bladder overactivity. The first group received 300 U BoNT-A and the second group received 200 U BoNT-A. The results were compared 4 and 16 weeks after injection: patients with idiopathic bladder overactivity responded to BoNT-A as well as those with neurogenic bladder overactivity and, despite the lower dose of toxin used, the results were similar.

Schmid et al. conducted a prospective study on 180 cases (135 women) to report the efficiency of a reduced dose (100 U) of BoNT-A injected into the bladder detrusor in case of patients with IOB [115]. Eighty-seven percent of patients experienced an improvement in urodynamic parameters: the urge completely disappeared in 75% of cases and the urinary incontinence disappeared in 84% of patients within two weeks. The frequency of urination decreased from 15 to 7 mictions and no more than 5 to 2 mictions per night were reported.

Brubaker et al. compared a group of 28 women with refractory urge through idiopathic bladder overactivity (200 U BoNT-A administered in the detrusor) with a placebo group of 15 women with the same pathology [116]. Sixty percent of the patients treated with BoNT-A have reported improved symptoms, effects that lasted approximately 373 days, respectively 62 days or less in the placebo cases. In the BoNT-A group, many patients with increased post-voiding residual volume (43%) and urinary tract infection were found in those with an increased post-voiding residual volume (75%).

Khanlow et al. conducted a study based on the effects of intravesical administration of BoNT-A in the management of refractory idiopathic bladder overactivity. Patients were properly informed about the improvement of life quality, the duration of re-injection and the risks for intermittent auto-catheterization. A cohort of 81 patients were injected with 200 U BoNT-A intravesically [117]. After BoNT-A injections, it was observed a significant improvement in the quality of life sustained by repeated injections. In 43% of cases treated with BoNT-A, auto-catheterization was required.

To describe the mid-term outcomes and adaptation of patients to BoNT-A therapy as a management strategy for women with refractory idiopathic bladder overactivity, Dowson et al. developed a cohort of 100 women who received BoNT-A injections as follows: all patients received an injection, 53 received 2, 20 received 3, 13 received 4, 10 received 5, 5 received 6, 3 received 7, 1 received 8, 1 received 9 and 1 received 10 injections [118]. Thirty-seven percent of patients completed the study after the first two injections and 11% of patients required intermittent auto-catheterization. As a possible complication, in 35% of patients, catheterization was required after the first administration of BoNT-A and in 21% of cases bacteriuria was detected. A review paper from 2016 investigated the use of BoNT in adults with urgency urinary incontinence and idiopathic overactive bladder. The conclusions of the study were that 22.9% to 55% cases regained complete continence and showed a significant improvement in the quality of life after treatment [119]. Detailed and comparative studies are found in Table 7.

## 4. Conclusions and Future Perspectives

In this paper, we reveal several pathologies from the gynecological field for which BoNT treatment can be used. Most of these dysfunctions have been shown to be refractory to conventional treatments, but the results after single or repeated cycles of BoNT-A were favorable and the symptoms improved.

The dosage used ranged from 40 U to 400 U in single administration. In some cases, repeated injection cycles were necessary, depending on the symptomatology and the scores obtained after the patients completed standardized questionnaires. The improvement of the symptomatology was objectively certified Susing standardized questionnaires before and after a certain period post-injection.

When compared to BoNT-B, we observed that BoNT-A is used more often with good results, especially because the paralysis resulting from BoNT-B is not as efficient as the one of BoNT-A. It is also desirable to carry out further studies to reach consensus on the optimal BoNT-A dose and administration protocol to create a standardized treatment.

In conclusion, this review highlights that BoNT could be successfully used in treating symptoms of gynecological dysfunctions refractory to conventional treatments, having few side effects and high efficacy.

## Figures and Tables

**Table 1:** Clinical uses of BoNT-A.

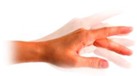	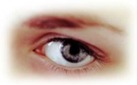	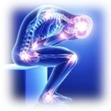	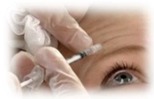	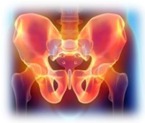	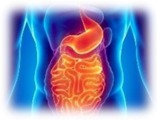	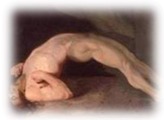
Neuromuscular Disorders	Ophthalmic Disorders	Chronic Pain	Cosmetic and Dermatological Applications	Pelvic floor Disorders	Gastrointestinal Disorders	Spasticity
Idiopathic/secondary focal dystonia	Misalignment	Tension headache	Wrinkles	Anismus	Achalasia	Stoke induced spasticity
Hemifacial Spasm/post-facial nerve palsy synkinesis	Paralytic strabismus	Cervicogenic headache	Face rejuvenation	Vaginismus	Bruxism	Cephalic tetanus
Tremor (essential, writing, palatal or cerebellar)	Therapeutic ptosis for corneal protection	Migraine	Hypersecretory disorders (hyperhidrosis, sialorrhea)	Detrusor sphincter dyssynergia	Temporomandibular joint dysfunction	Multiple sclerosis
Tic disorders	Restrictive or myogenic strabismus	Lower back ache	Glabellar frown	Chronic anal fissures	Palatal myoclonus	Traumatic brain injury
Myokymia	Upper eyelid retraction	Tennis elbow	Vertical platysma bands	Perineal muscles spasm	Esophageal diverticulosis	Cerebral palsy
Neuromyotonia	Duane’s syndrome	Myofascial pain	Browlift	Vulvodynia	Laryngeal disorders	Spinal cord injury

**Table 2:** Classification of BoNTs.

Origin		BoNT Serotype	Target Substrate	Bont Subtype	Substrate Localization
C. *Botulinum* group I		A	SNAP-25	A1; A2; A3; A4; A5; A6; A7; A8; A9; A10; A(B); Ab; Af; Af84	Presynaptic plasma membrane
		B	VAMP	B1; B2; B3; B5(Be); B6; B7; Bf	Synaptic vesicle
		F	VAMP_1_, VAMP_2_	F1; F2; F3; F4; F5	Synaptic vesicle
		X	VAMP_4_, VAMP_5_, Ykt6	-	Synaptic vesicle
C. *Botulinum* group II		B	VAMP	B4	Synaptic vesicle
		E	SNAP 25	E1; E2; E3; E6; E7; E8; E9; E10	Presynaptic plasma membrane
		F	VAMP_1_, VAMP_2_	F6	Synaptic vesicle
C. *Botulinum* group III		C	SNAP 25, Syntaxin 1A, Syntaxin 1b	C; CD;	Presynaptic plasma membrane
		D	VAMP_1_, VAMP_2_	D; DC	Synaptic vesicle
C. *Botulinum* group IV (*C. argentinese*)		G	VAMP_1_, VAMP_2_	G	Synaptic vesicle
Other organisms producing BoNTs	C. *Butyricum*	E	SNAP 25	E4; E5	Presynaptic plasma membrane
	C. *Baratii*	F	VAMP_1_, VAMP_2_	F4	Synaptic vesicle
	*Enterococcus faecium strain*	En	VAMP_2,_ SNAP25	-	Synaptic vesicle
	*Weissella oryzae* SG25T	Wo	VAMP_2_	-	Synaptic vesicle

**Table 3:** Studies of BoNT-A in the treatment of vaginism.

Author	Study Design	Number of Cases	Treatment Regimen	Outcome Measures	Follow-Up	Results
Ghazizadeh [54]	Retrospective study	24	Dilution: 500 U of BoNT-A diluted with 1.5 mL of normal saline solution. Dose: 150–200 U injected first; the dose gradually increased the total dose of 400 U * Dysport, Ipsen Ltd., Maidenhead, UK	Vaginal muscles resistance	12.37 months	23 patients had vaginal examinations 1-week post injection that showed little or no vaginismus 18 patients achieved satisfactory intercourse after the first injection 4 patients had mild pain 1 patient needed a second injection; 1 patient refused vaginal examination and did not attempt to have coitus
Shafik [55]	Case-control study	13	BoNT group: A single injection; dose and dilution: 25 U diluted in 1 mL saline solution Control group: saline solution	Satisfaction of intromission	3.3 months	All the symptoms at patients injected with BT improved. There was no recurrence during the follow-up period Control subjects did not improve
Bertolasi [56]	Prospective study	39	Repeated cycles at 4 weeks of botulinum neurotoxin injected into levator ani. * Dysport, Ipsen Ltd., Maidenhead, UK	Possibility of sexual intercourse; levator ani EMG hyperactivity; Lamont scores, VAS, FSFI	105 (±50 SD) weeks	At 4 weeks after the first cycle the primary outcome improved, as did the secondary outcomes When follow-up ended, 63.2%—were completely recovered; 15.4% still needed reinjections and 15.4% had dropped out the study
Pacik [57]	Retrospective study	20	Dose: 100 to 150 U of BoNT-A; Dilution: 100 U of BoNT-A diluted in 2 mL of saline; * Allergan, Inc., Irvine, CA, USA.	Possibility of having intercourse	Time of follow up not reported	80% of patients achieved intercourse in maximum 3 months 15% of patients continued the injections (maximum 6 dilators); 5% of patient did not respond to treatment (unable to advance beyond the first dilator)
Pacik [59]	Clinical trial	241	Dose: 100 U of BoNT-A; Dilution: 2 mL of saline; * onabotulinumtoxinA; Allergan, Irvine, CA, USA	Pain and anxiety scores; time to achieve intercourse untoward effects.	1 month, 3 months, 6 months, 1 year	71% reported at a median of 2.5-week pain-free intercourse; 2.5% were unable to achieve intercourse during follow up 26.6% were lost within 1 year after treatment. 1.24% developed mild temporary stress incontinence, 0.41% temporary excessive vaginal dryness

**Table 4:** Studies of BoNT-A in treatment of vulvodynia.

Author	Study Design	Number of Cases	Treatment Regimen	Outcome Measures	Follow-Up	Results
Yoon [60]	Retrospective study	7	Dilution: 20 U of the BoNT diluted in saline solution; Dose: 20 U of BoNT-A * Botox, Allegran, Inc., Irvine, CA, USA	VAS (before and 2 weeks after each administration)	4–24 months	The subjective pain score improved from 8.3 to 1.4, and no one has experienced a recurrence. No adverse effects were observed; In 2 cases, pain decreased after one injection; 5 cases needed injections twice; Patients reported subjective improvement in sexual; life and having no significant pain or discomfort during or after intercourse.
Petersen [63]	Randomized, double blinded, placebo-controlled study	32 cases 32 placebo	Dilution: 100 U of BoNT-A diluted in 2.5 mL saline solution; Dose: 20 U of the BoNT diluted or 0.5 mL of saline (placebo) * Botox, Allergan	VAS, FSFI; FSDS; Manifest Female Sexual Dysfunction; Demographic Questionnaire; SF-36	3, 6, 9, and 12 months	Both groups: significantly pain reduction (*p* < 0.001); No significantly improvements on the FSFI score until the second follow up visit (*p* = 0.635); Compared to the group treated with BoNT-A, in the placebo group it was observed higher decrease of the sexual distress until the second follow-up (*p* = 0.044).
Pelletier [66]	Retrospective study	20	Dilution: 1 mL: 50 U BoNT-A diluted in 1 mL saline; Dose: 50 UIBoNT diluted * Botox; Allergan, Courbevoie, France	VAS; FSFI; DLQI	3, 6 months	16 patients reported improved VAS scores; At the 3 months follow up visit, 13 patients reported possibility of sexual intercourse; After the 6 months follow up visit, QoL and sexual function reported to be satisfactory.
Jeon [67]	Retrospective study	73	Dose: 40 to 100 U BoNT-A (11 patients) 300 to 600 mg Gabapentin (62 patients) * Botox, Allegran Inc., Irvine, CA, USA	VAS	6 to 24 months	Gabapentin group: the VAS score decreased from 8.6 to 3.2 after treatment (*p* < 0.001); BoNT-A group: the VAS score decreased from 8.1 to 2.5 (*p* < 0.001).

**Table 5:** Studies of BoNT-A in chronic pelvic pain and pelvic floor muscle spasm treatment.

Author	Study Design	Number of Cases	Treatment Regimen	Outcome Measures	Follow-Up	Results
Adelowo [73]	Retrospective cohort study	31	Dose: 100–300 U BoNT-A * Botox, Allergan Inc. Irvine, CA, USA	Patient-reported tenderness on levator palpation; patient-reported symptom improvement; time to and number of repeat injections; complications	<6 weeks post-injection (visit 1) and ≥6 weeks post injection (visit 2).	93.5% completed the first follow-up visit; 79.3% reported improvement in pain and 20.7% reported no improvement. Median pain with levator palpation was significantly lower than before injection (*p* < 0.0001). 58.0% had a second follow-up visit with a median pain score lower than before injection (*p* < 0.0001); the median time to repeat injection was 4.0 (3.0–7.0) months; 10.3% women—de-novo urinary retention, 6.9%-fecal incontinence, 10.3%-constipation and/or rectal pain; all side effects resolved spontaneously.
Abott [74]	Double-blinded, randomized, placebo-controlled trial.	60	Cases: 80 U BoNT-A (20 units/mL) Placebo: 4 mL of saline solution * Botox, Allergan Westport, Ireland	Dysmenorrhea; dyspareunia; dyschezia; Non-menstrual; pelvic pain assessed VAS scale	0, 1, 2, 3, 4, 5 and 6 months	In case of dyspareunia and non-menstrual pain, it was observed that VAS score improved in the group treated with BoNT (66 vs. 12 and 51 vs. 22 respectively; also, the pelvic floor pressure decreased (49 vs. 32) Dyspareunia was reduced in the placebo group (64 vs. 27);
Jarvis [77]	Prospective study	12	Dose: 40 U BoNT; Dilutions: 10 U/mL; 20 U/mL; and 100 U/mL. * Allergan (Gordon, New South Wales, Australia).	VAS; SF-12; EQ-5D; Pelvic floor muscles manometry; Sexual activity scores	2, 4, 8 and 12 weeks post-treatment	In case of dyspareunia and dysmenorrhea, VAS scores improved (80 vs, 28; *p* = 0.01, respectively 67 vs. 28; *p* = 0.03). SF-12, EQ-5D and sexual activity scores were improved until week 12.
Morrissey [78]	Prospective pilot open-label study	21	Dose: up to 300 U BoNT-A Administration: using needle electromyography guidance, from a transperineal approach, to localize spastic pelvic floor muscles * Botox; Allergan, Irvine, CA, USA	VAS scores for pain and dyspareunia; QoL and sexual function; GRA scale for pelvic pain; pelvic floor tone and tenderness; vaginal manometry.	6 months (4, 8, 12, and 24 weeks after injections)	61.9% of subjects reported improvement on GRA at 4 weeks and 80.9% at 8, 12, and 24 weeks post injection, compared with baseline; 58.8%, 68.8%, 80% and 83.3% reported less dyspareunia at 4, 8, 12, and 24 weeks, respectively. VAS score improved at weeks 12 (5.6, *p* = 0.011) and 24 (5.4, *p* = 0.004) compared with baseline (7.8); Sexual dysfunction as measured by the FSDS significantly improved; SF-12 showed improved QoL in the physical composite score at all post injections visits (42.9, 44, 43.1, and 45.5 vs 40 at baseline; *p* < 0.05), Vaginal manometry—decrease in resting pressures and in maximum contraction pressures at all follow-up visits (*p* < 0.05); Digital assessment of PFM showed decreased tenderness on all visits Reported post-injection adverse effects: worsening of the following preexisting conditions: constipation (28.6%), stress urinary incontinence (4.8%), fecal incontinence (4.8%), and new onset stress urinary incontinence (4.8%).
Rao [80]	Randomized, placebo-controlled study	12	Cases: 100 U of BoNT-A intra sphincterian (anal) at 3 months intervals; Placebo: saline solution * Botox; Allergan Pharmaceuticals, Los Angeles, CA, USA)	Daily frequency; VAS; balloon expulsion; anorectal manometry, pudendal nerve latency tests	NR	The VAS score did not improve (*p* = 0.31) compared with baseline; At 3 months follow up, the mean VAS pain score was decreased: 6.79 vs. 7.08 (*p* = 0.25); Rectal sensory thresholds, anal sphincter pressures, balloon expulsion times, pudendal nerve latency did not decrease after BoNT-A or placebo

**Table 6:** Studies of BoNT in interstitial cystitis.

Author	Study Design	Number of Cases	Treatment Regimen	Outcome Measures	Follow-Up	Results
Pinto [93]	Prospective study	17	Dose: 100 U of Botulin toxin Administration: bladder trigone only, under cystoscopy guidance * Botox (Allergan, Inc., Irvine, CA, USA)	3-day voiding chart; VAS O’Leary-Sant score pressure flow study and flowmetry	9 months	Pain score decreased at 1 month follow up visit and 3 months follow up visit (from 5.7 to 2.2 and 1.9) (*p* < 0,01); At the end of the study, 41.17% of patients reported increased urinary frequency with lower threshold of pain and O’Leary-Sant score; All patients reported subjective improvement.
Giannantoni [94]	Prospective Study	7	Dose: 200 U BoNT-A, diluted in 100 mL saline, without any form of anesthesia. Administration: intravesical instillation, retained in the bladder for 40 mi * Botox (Allergan, Inc., Irvine, CA, USA)	voiding diary; urodynamic; Visual Analog Scale for pain assessment	3 months	At baseline mean day- and night-time urinary frequencies were 9.1 and 4.6, respectively. Mean VAS score was 6.5. On urodynamics, mean bladder capacity was 270.4 mL. No patients showed any impairment of bladder emptying 1 week after treatment, mean day and night-time urinary frequency fell to 7.4 and to 3.3; VAS score significantly dropped to 3.5 (*p* < 0.05). VAS score improvement was particularly marked in 4 patients Maximum cystometric capacity was 321.4 mL. Symptoms and urodynamic parameters did not change in 3/7 patients; No local or systemic side effects were reported during or after instillation
Giannantoni [95]	Prospective study	15	Dose: 200 U BoNT-A diluted in 20 mL saline; * Botox (Allergan, Inc., Irvine, CA, USA)	3-day voiding chart; pain visual analog scale; urodynamics	12 months	at follow up visit from 1 and 3 months, 86.6% of patients reported improvement in the symptoms; decreased urinary frequency and VAS score; At the last follow up visit all patients reported re-apparition of pain; Complication: in 9 cases after 1 month, 4 cases at the 3-month visit and in 2 cases at 5-month visit, the patients reported dysuria.
Kuo [96]	Prospective study	10	Dose: In 5 patients, 100 U of BoNT-A; additional 100 U BoNT-A into the trigone in the other 5 patients. Administration: suburothelial into 20 sites * Botox, Allergan Inc. Irvine, CA, USA	number of daily urinations; urodynamic changes functional bladder capacity; bladder pain;	3 months	functional bladder capacity significantly increased (155 after injection vs. 77 mL at baseline, *p* < 0.001) VAS scores and frequency of daily urinations were decreased urinary frequency and bladder pain were improved after the 3 months follow up visit in 2 patients The urodynamic results (cystometric capacity) were improved (287 vs. 210 mL, *p* = 0.05).
Carl [97]	Two center pilot study	29	Dose:500 U BoNT-A diluted in 3 mL saline Administration: injected through a rigid cystoscope into 20–25 sites submucosally in the trigone and bladder floor. * Dysport^®^ (Ipsen Pharma, Ettlingen, Germany	Daytime frequency; nycturia; urgency; Pain (VAS score) Urodynamic evaluation	6 months	Daytime frequency, nycturia, urgency and pain by VAS scale decreased by 50%, 75%, 43% and 81%, respectively, 6 weeks after treatment (*p* < 0.05); maximal cystometric capacity increased from 282 to 360 mL; bladder compliance increased from 13 mL/cmH_2_0 to 23 mL/cmH_2_O; Two patients suffered from temporary hematuria, 3 patients had residual urine of more than 100 cc and 1 patient showed urinary retention
Ramsay [98]	Prospective study	11	Dose: 200–300 U-BoNT-A; Administration: BoNT was injected in 20–30 different sites (10 U per site) into the suburothelium of the bladder * source of toxin not reported	BFLUTS; KHQ; 24-h voiding frequency chart; Filling and voiding urodynamics; Urodynamic variables PIP1	14 weeks	Baseline BFLUTS score 132.Improved to 118 at 6 weeks (19%, *p* = 0.07); improvement was maintained at 10 weeks (score=109, 22%, *p* = 0.03) and 14 weeks (score=105, 27%, *p* = 0.01); Frequency improved post injection to 12 at 6 weeks (*p* = 0.57), 10 at 10 weeks (*p* = 0.01) and 10.5 at 14 weeks (*p* = 0.03); FDV improved from 96 to 174 mls (*p* = 0.04); MCC improved from 200 to 258 mls (*p* = 0.16); BFLUTS improved at 6 weeks (*p* = 0.009) and 10 weeks (*p* = 0.009); PIP1 increased from 39 to 46 (*p* = 0.9).
Pinto [99]	Prospective study	16	Dose: 100 U BoNT-A Administration: 4 consecutive injections of BoNT-A injected intratrigonal under cystoscopic guidance * Botox (Allergan, Inc., Irvine, CA, USA)	VAS Voiding dysfunction; O’Leary-Sant score; Urinary tract infections	12 months	VAS score and O’Leary-Sant score decreased; urinary frequency increased; quality of life scores was similar after each injection; The effects of BoNT-A lasted an average of 9.9 ± 2.4 months.

**Table 7:** Studies of BoNT in overactive bladder.

Author	STUDY DESIGN	Number of Cases	Treatment Regimen	Outcome Measures	Follow-Up	Results
Le Normand [113]	Prospective, randomized, double-blind, placebo-controlled comparative study	99	Dose: 50 U, 100 U or 150 U BoNT-A Administration: intradetrusor injection * Botox (Allergan, Inc., Irvine, CA, USA)	Clinical and urodynamic variables; Quality of life (QoL)	day 8; 1, 3, 5, and 6 months	after three months >50% improvement in urgency and urge urinary incontinence in 65% and 56% of patients who respectively received 100 U (*p* = 0.086) and 150 U (*p* = 0.261) BoNTA—>75% improvement in 40% of patients of both groups (100 U [*p* = 0.058] and 150 U [*p* = 0.022]); Complete continence: in 55% and 50% patients after 100 UI and 150 U BoNTA treatment at month 3; QoL improved up to the 6-month visit; 3 patients with postvoid residuals >200 mL in the 150 U group and a few urinary tract infections.
Popat [114]	Prospective, open label study	75	Dose: 300 U (NOB) or 200 U (IOB) of BoNT Administration: injected into the bladder * Botox (Allergan, Inc., Irvine, CA, USA)	urodynamic maximum cystometric capacity; maximum detrusor pressure during filling; number of incontinence episodes; frequency of voids;	1 month and 4 months	At 4 months, cystometric capacity increased in NOB and IOB cases treated with BoNT (229.1 to 427.0 mL, *p* < 0.0001, respectively 193.6 to 327.1 1 mL, *p* = 0.0008; Decreased maximum detrusor pressure during filling in NOB and IOB (60.7 to 26.1 cm H_2_O, *p* <0.0001, respectively 62.1 to 45.1 cm H_2_O, *p* = 0.027); Frequency decreased in NOB and IOB patients (12.3 to 6.6 voids/24 h, *p* <0.0001, respectively 13.6 to 8.3, *p* = 0.0002); Urgency decreased in NOB and IOB (7.5 to 1.44 episodes/24 h, *p* <0.0001, respectively 10.9 to 4.9, *p* < 0.0001)
Schmid [115]	Prospective study	180 (45 men, 135 women)	Dose: 100 U of BTX-A into the detrusor at 30 different sites. Reinjection: 52/180 of patients were reinjected after the effect had diminished (time interval between two treatments was mean 11 months) * Botox (Allergan, Inc., Irvine, CA, USA)	Urgency, frequency, maximal cystometric capacity (MCBC) volume at first and strong desire to void (FDV, UV) detrusor compliance (DC), postvoiding residual volume (PVR), QoL assessment	After 4, 12 and 36 weeks	87% of patients showed a significant (*p* > 0.001) improvement of their bladder function urgency completely disappeared in 75% and incontinence in 84% within 2 weeks; frequency decreased from 15 to 7 micturition and nycturia from 5 to 2; MCBC increased from mean 245 to 395 mL; FDV increased from mean 127 to 218 mL; strong desire to void from mean 215 to 312 mL; QoL assessment revealed a significant subjective improvement in all urge-related items; side effects: 6 temporary urine retentions and 16 urinary infections.
Brubaker [116]	Randomized, double-blind, placebo controlled, review	43	Dose: 200 U BoNT dissolved in 6 mL saline Placebo: 3 mL saline. * Botox (Allergan, Inc., Irvine, CA, USA)	frequency of incontinence episodes; symptom and quality of life measures (PGISC); the duration and occurrence of voiding dysfunction	12 months	60% of the cases injected with BoNT-A reported improved PGISC scores post-void residual urine increased in 43% of cases urinary tract infection rate increased in the cases with increased post-void residual urine
Khanlow [117]	Prospective, open label study	81	Dose: 200 U BoNT-A Administration: intradetrusor injections at 20 sites per injection * Botox (Allergan, Inc., Irvine, CA, USA)	UDI IIQ	NR	Mean UDI and IIQ scores improved after injection 1 in all patients (56 to 26 and 59 to 21), after injection 2 in 29.6% of cases (52 to 30 and 51 to 24), after injection 3 in 16.04% of cases (40 to 19 and 43 to 17), after injection 4 in 7.40% (44 to 17 and 61 to 15) and after injection 5 in 4.93% (51 to 17 and 63 to 14). In 43% of cases, self-catheterization was requested
Dowson [118]	Prospective study	100	Dose: 200 U BoNT-A Administration: into suburothelium or detrusor muscle under cystoscopic guidance * Onabotulinumtoxin A; Allergan Ltd., Marlow, Buckinghamshire, UK	QoL measures; voiding diary; residual volume; complications	To five BoNT-A injections.	37% of patients completed the study after the second administration of BoNT-A (13% of cases reported poor efficacy and 11% due to the need of intermittent self-catheterization) In 35% of cases, the need of self-catheterization was seen after the first administration of BoNT-A. The period between administration of BoNT doses was ~322 days

## Abbreviations

The following abbreviations are used in the manuscript:

*Ach*	acetylcholine
*BFLUTS*	Bristol female low urinary tract symptoms
*BoNT*	botulinum toxin
*BoNT A*	botulinum toxin type A
*BoNT B*	botulinum toxin type B
*CISC*	intermittent self-catheterization
*DC*	detrusor compliance
*EMG*	electromyography
*EQ 5D*	a standardized instrument for use as a measure of health outcome
*FSDS*	female sexual distress scale
*IIQ*	Incontinence inventory questionnaire
*IOB*	idiopathic overactive bladder
*KHQ*	King’s Health Questionnaire
*LC*	Light chain
*MCBC*	maximal cystometric capacity
*NGF*	nerves growth factor
*NOB*	neurogenic overactive bladder
*OB*	overactive bladder
*PFM*	pelvic floor muscle
*PGISC*	patient global impression of symptom control
*PVR*	postvoiding residual volume
*QoL*	quality of life
*SNARE*	Soluble NSF(N-ethylmaleimide-sensitive factor) Attachment Protein) REceptor
*SF12*	physical and mental health summary scales
*SNAP*	Synaptosomal-associated protein 25
*TRPV*	transient receptor potential cation channels (“V” is for vanilloid type)
*UDI*	urinary distress inventory
*VAS*	visual analog scale
*VAMP*	vesicle associated membrane protein
